# Patient-reported outcomes version of the common terminology criteria for adverse events and quality-of-life linear analogue self-assessment in breast cancer patients receiving radiation therapy: single-institution prospective registry

**DOI:** 10.1186/s41687-021-00408-9

**Published:** 2022-01-10

**Authors:** C. S. Thorpe, T. A. DeWees, M. A. Golafshar, R. S. Bhangoo, T. Z. Vern-Gross, L. A. McGee, W. W. Wong, M. Y. Halyard, S. R. Keole, C. E. Vargas

**Affiliations:** 1 Department of Radiation Oncology, Mayo Clinic, 5881 E Mayo Blvd., Phoenix, AZ USA; 2 Department of Health Sciences Research, Mayo Clinic, Scottsdale, AZ USA

## Abstract

**Purpose/objectives:**

We sought to investigate the impact of patient-reported outcomes version of the common terminology criteria for adverse events (PRO-CTCAE) on overall quality-of-life (QOL) employing linear analogue self-assessment (LASA) in breast cancer (BC) patients undergoing radiation therapy (RT).

**Materials/methods:**

All patients treated with RT for BC with curative intent from 2015 to 2019 at our institution were included. Breast specific PRO-CTCAE and overall QOL LASA questionnaires were administered at baseline, end-of-treatment, 3, 6, 12 months, and then annually. Minimal clinically important difference in overall QOL was a 10-point change in LASA. Hypofractionation was any treatment > 2 Gy per fraction. Mixed models for repeated measures were used to determine the association of PRO-CTCAE and overall QOL LASA.

**Results:**

Three hundred thirty-one (331) patients with a median follow-up of 3.1 years (range 0.4–4.9) were included. Average overall QOL LASA scores were 78.5 at baseline, 79.8 at end-of-treatment, 79.8 at 3 months, 77.1 at 6 months, 79.4 at 12 months, and 79.7 at 24 months. On univariate analysis, patients reporting a grade ≥ 3 PRO-CTCAE had, on average, a 10.4-point reduction in overall LASA QOL (p < 0.0001). On multivariate analysis, not being treated with hypofractionation and higher BMI were predictive for worse overall LASA QOL with a 10-point reduction in LASA for patients reporting a grade ≥ 3 PRO-CTCAE (p < 0.0001).

**Conclusions:**

Patients reporting a grade ≥ 3 PRO-CTCAE experienced statistically significant and clinically meaningful deterioration in overall QOL LASA. Hypofractionation improved QOL while higher BMI predicted for worse QOL. PRO-CTCAE should be integrated into future clinical trials.

## Introduction

Adverse events during cancer treatment are recorded using the US National Cancer Institute’s (NCI) Common Terminology Criteria for Adverse Events (CTCAE) [1]. However, symptomatic adverse events are often underreported by physicians and do not fully represent the entirety of the negative impact that cancer treatment has on patients [2, 3].

As a result, patient reported outcomes (PROs) have been developed and provide complimentary information to physician reported measures [4]. In fact, PROs have been shown to predict for overall survival for many different cancers [5–7]. There are numerous tools to measure PROs including the NCI’s Patient-Reported Outcomes version of the Common Terminology Criteria for Adverse Events (PRO-CTCAE) [8]. This was developed by a multidisciplinary group of investigators who identified 78 symptomatic adverse events (AEs) in CTCAE that were deemed to be appropriate for patient reporting for patients undergoing cancer treatment including surgery, chemotherapy, and/or radiation therapy [8]. Investigators then came up with plain language terms corresponding to these items. Each of the items was also assessed to determine the applicability to rate according to frequency, severity, and/or activity interference for the patient. This resulted in 124 items in the PRO-CTCAE each graded on a 5-point scale corresponding to the CTCAE scale [8]. The answer choices for items grading frequency include Never, Rarely, Occasionally, Frequency, and Almost constantly. Severity response options include None, “Mild”, Moderate, Severe, and Very severe. Lastly, interference item responses include Not at all, A little bit, Somewhat, Quite a bit, and Very much. PRO-CTCAE has proven to be valid and reliable compared to other measures [9].

PROs can also be assessed with single item measures such as a numerical linear analogue self-assessment (LASA) which have been shown to be valid in assessing overall quality-of-life in cancer patients [10, 11]. Single item measures (e.g. LASA) offer potential advantages over multi-item measures (e.g. PRO-CTCAE) including brevity, simplicity, minimal time commitment, limited additional burden on the patient, and ease of use [11, 12]. However, single item measures lack the ability to identify precisely what factors are contributing to the score. Thus, multi-item scores are complimentary to single item measures and may be able to identify contributing factors to the overall measure [6].

This analysis reports on the relationship between PRO-CTCAE and overall quality-of-life using LASA in breast cancer patients undergoing curative intent radiation.

## Methods and materials

We included all patients treated with curative intent radiation therapy for breast cancer at our institution from 2015 to 2019. The study was approved by the Institutional Review Board and informed consent was obtained from each patient to be included on the institutional prospective registry. Patients underwent breast-conserving surgery or mastectomy. Chemotherapy, if indicated, was administered either in the neoadjuvant or adjuvant setting. Radiation was delivered with either photons or proton beam therapy at the discretion of the treating physician. Target volumes included the whole breast or chest wall with or without regional nodes at the discretion of the treating physician. Treatment was delivered as conventional fractionation (1.8–2 Gy/fraction) or hypofractionation (> 2 Gy per fraction).

Our institution had previously developed breast-specific PRO questionnaires including items from The Breast Cancer Treatment Outcomes Scale, PRO-CTCAE, Patient-Reported Outcomes Measurement Information System (PROMIS), and LASA that were routinely administered to all breast cancer patients undergoing radiation. The questionnaires were administered to all patients via an online portal or at radiation oncology office visits via tablet at baseline before radiation, end of radiation, 3 months, 6 months, and 12 months after radiation, and then annually. Patients were required to have baseline questionnaires and at least 1 other time point to be included. The current study only looked at the PRO-CTCAE items and overall QOL LASA. The PRO-CTCAE items that were asked on the institution derived questionnaire included anxiety interference, insomnia interference, severity of shortness of breath, shortness of breath interference, severity of cough, concentration interference, sad interference, severity of skin burns, severity of difficulty swallowing, decreased appetite interference, how often nausea, severity of constipation, numbness or tingling in hands or feet interference, and how often loose or watery stools (diarrhea). A patient was considered to have patient-reported treatment-related symptomatic adverse event at each time-point, if she had a score of 3 or higher (‘severe’ or worse) for any breast specific PRO-CTCAE item that was higher than baseline. Overall LASA was scored on the 0–10 scale and transformed to a continuous 0–100 point scale. Minimal clinically important difference in overall QOL is defined, herein, as a 10-point decrease in LASA transformed score [13].

### Statistics

Mixed models for repeated measures, controlled for baseline LASA score, was utilized to model the association between overall QOL LASA scores with PRO-CTCAE, socio-demographic, clinical, and treatment covariates. Univariate (UVA) models are presented, and multivariate models were determined utilizing statistically and clinically significant factors to determine ‘best’ model based on Bayes’ Information Criteria (BIC).

## Results

Three hundred thirty-one (331) patients with a median follow-up of 3.1 years (range 0.4–4.9) were included. Patient and tumor characteristics are listed in Table 1. Most patients were pT1 (51%), pN0 (64%), ER + (82%), PR + (77%), and had invasive ductal carcinoma (85%). Treatment characteristics are listed in Table 2. Patients were typically treated with lumpectomy (75%) followed by whole breast radiation to 40.05 Gy. Most patients (85%) were treated with photons. Three hundred thirty-one patients answered questionnaires at baseline, 255 at end of treatment, 205 at 3 months after treatment, 214 at 6 months after treatment, 128 at 12 months after treatment, and 34 at 24 months after treatment.

**Table 1:** Patient and tumor characteristics

	n = 331
Age, years (range)	60 (29–87)
BMI (range)	26.8 (17.3–50.7)
ECOG	
0	219 (66%)
1	108 (33%)
2	4 (1%)
Laterality	
Right	163 (49%)
Left	168 (51%)
Histology	
Invasive ductal	283 (85%)
Invasive lobular	13 (4%)
Mixed	5 (2%)
Other	30 (9%)
Grade	
1	71 (21%)
2	148 (45%)
3	111 (34%)
Not reported	1 (< 1%)
pT stage	
T0	25 (8%)
Tis	52 (16%)
T1	169 (51%)
T2	63 (19%)
T3	21 (6%)
Not reported	1 (< 1%)
Nodal status	
Negative	211 (64%)
Positive	87 (26%)
Not reported	33 (10%)
ER positive	271 (82%)
PR positive	255 (77%)
HER2 positive	33 (10%)
TNBC	27 (8%)

BMI, body mass index; ECOG, Eastern Cooperative Oncology Group; ER, estrogen receptor; PR, progesterone receptor; TNBC, triple negative breast cancer

**Table 2:** Treatment characteristics

Type of surgery	
Lumpectomy	249 (75%)
Mastectomy	48 (15%)
Other	34 (10%)
Sentinel lymph node biopsy	223 (67%)
Axillary lymph node dissection	71 (21%)
Any chemotherapy	129 (39%)
Radiation dose median, Gy (range)	40.05 (40.05–50.4)
Radiation boost median, Gy (range)	10 (5.4–10.5)
Received boost	131 (40%)
Nodal radiation	48 (15%)
Hypofractionation*	247 (75%)
Treated with photons	281 (85%)
Treated with protons	50 (15%)

^*^Hypofractionation defined as any treatment > 2 Gy per fraction

PRO-CTCAE of grade ≥ 2 (‘moderate’ or worse) above baseline were reported by 75% of patients and grade ≥ 3 (‘severe’ or worse) above baseline were reported by 32%. Specific PRO-CTCAE items grade ≥ 2 or ≥ 3 above baseline at any time are listed in Table 3. The most common grade ≥ 2 items were dermatitis (76%), insomnia (40%), and anxiety (32%). The most common grade ≥ 3 items were dermatitis (33%), insomnia (28%), and concentration (28%).

**Table 3:** PRO-CTCAE Grade ≥ 2 and ≥ 3 at any time

Question	PRO-CTACE Grade ≥ 2
Anxiety	108 (32%)
Appetite	27 (8%)
Concentration	104 (31%)
Cough	98 (31%)
Insomnia	133 (40%)
Interfere with breath	30 (9%)
Nausea	52 (16%)
Sadness	98 (30%)
Severity of breath	97 (30%)
Skin	204 (76%)
Swallowing	54 (18%)
Tingling	40 (12%)

	PRO-CTCAE Grade ≥ 3
Anxiety	51 (15%)
Appetite	9 (3%)
Concentration	32 (28%)
Cough	33 (10%)
Insomnia	91 (28%)
Interfere with breath	13 (4%)
Nausea	19 (6%)
Sadness	83 (25%)
Severity of breath	60 (19%)
Skin	87 (33%)
Swallowing	20 (7%)
Tingling	18 (6%)

PRO-CTCAE, Patient-Reported Outcomes version of the Common Terminology Criteria for Adverse Events

**Fig. 1:**
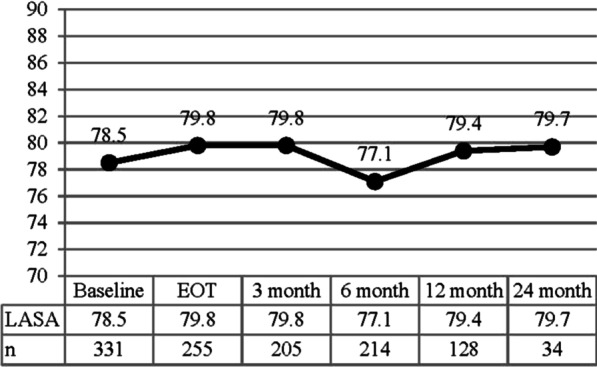
LASA over time. LASA at each timepoint did not change significantly over time (p = 0.7). EOT, end of treatment

**Fig. 2:**
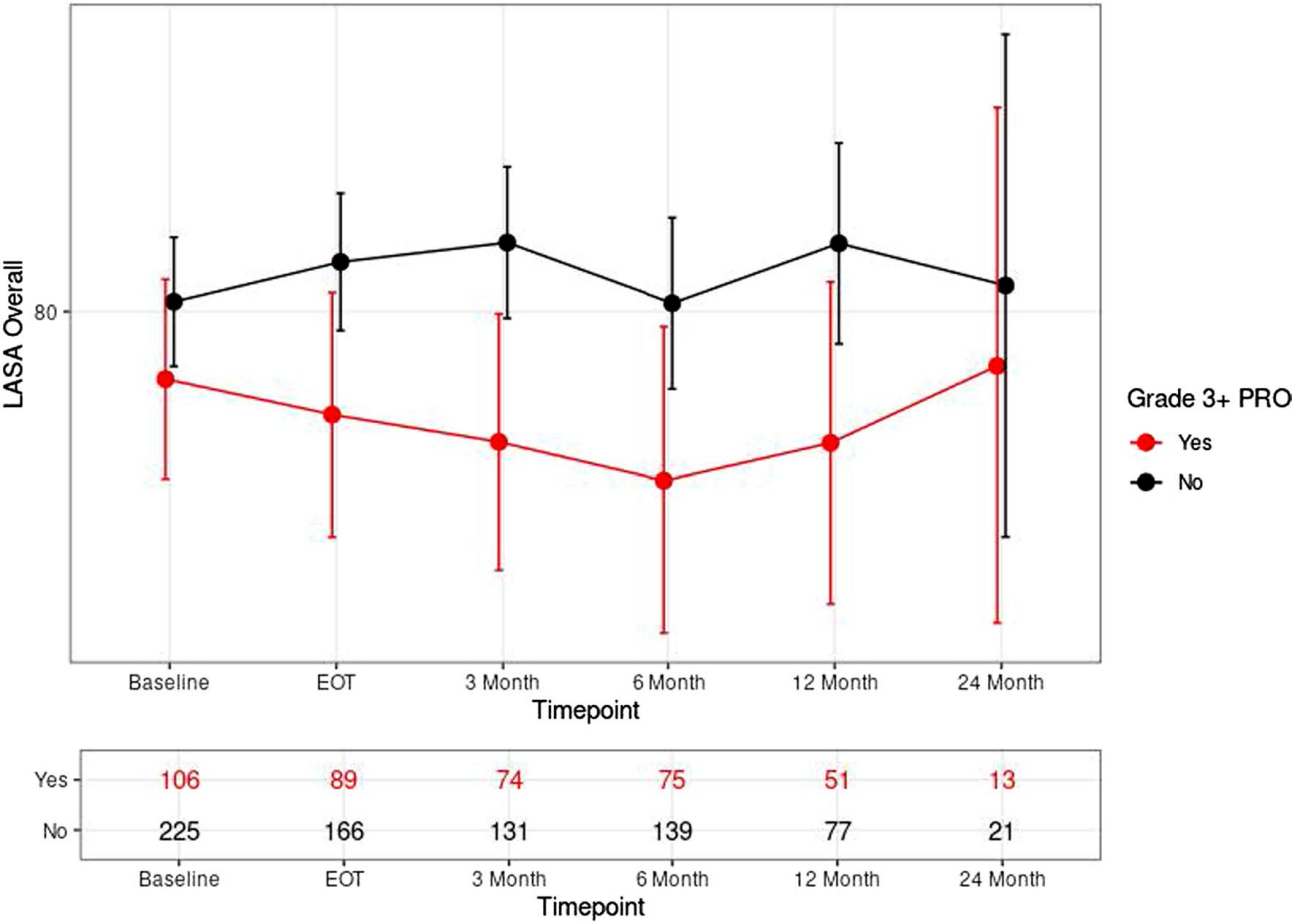
Overall QOL LASA and Grade ≥ 3 PRO-CTCAE over time. Overall average QOL LASA at each time point according to patients who reported a grade ≥ 3 (‘severe’ or worse) PRO-CTCAE. Number of patients at each time point is found below the graph. EOT, end of treatment; PRO, patient-reported outcomes

Average overall QOL LASA scores for all patients did not change significantly over time (p = 0.7). Scores were 78.5 at baseline, 79.8 at end of treatment, 79.8 at 3 months, 77.1 at 6 months, 79.4 at 12 months, and 79.7 at 24 months (Fig. 1). On univariate analysis, increasing BMI and patients who experienced a grade ≥ 3 (‘severe’ or worse) PRO-CTCAE reported significantly lower overall QOL LASA score (p ≤ 0.0001 for both) (Table 4). Additionally, patients treated with hypofractionation had improved (higher) overall QOL LASA scores (p = 0.004). On multivariate analysis, hypofractionation improved overall QOL LASA (4.08 points, p = 0.004) and higher BMI led to worse overall QOL LASA (− 0.58 points per 1 kg/m^2^ increase in BMI, p < 0.0001). Importantly, patients who reported grade ≥ 3 (‘severe’ or worse) PRO-CTCAE were found to have a 10-point drop in their overall QOL LASA score (p < 0.0001) (Fig. 2).

**Table 4:** Univariate and multivariate analysis showing estimates of LASA point changes

Variable	UVA estimate (95% CI)	P value	MVA estimate (95% CI)	P value
Age	0.11 (− 0.002 to 0.22)	0.055		
Body mass index	− 0.58 (− 0.77 to − 0.34)	< 0.0001	− 0.5515 (− .74 to − 0.36)	< 0.0001
Node Positive	− 1.93 (− 4.71 to 0.85)	0.174		
Sentinel lymph node biopsy	− 2.14 (− 4.63 to 0.34)	0.091		
Axillary lymph node dissection	− 0.44 (− 3.36 to 2.48)	0.766		
Boost	1.59 (− 0.8 to 3.99)	0.191		
Hypofractionation	4.08 (1.35–6.81)	0.004	3.6488 (1–6.3)	0.007
Proton	1.14 (− 2.05 to 4.34)	0.482		
PRO-CTCAE Grade ≥ 3	− 10.41 (− 13.57 to − 7.25)	< 0.0001	− 10.02 (− 13.13 to − 6.91)	< 0.0001

CI, confidence interval

## Discussion

We compared PROs using LASA and PRO-CTCAE measures for breast cancer patients receiving radiation treatment in a prospective institutional registry. Our overall QOL LASA scores are consistent with other reports [11, 14]. This study supports the use of single item PROs, like LASA, for breast patients receiving radiation therapy. Patients who reported a PRO-CTCAE grade ≥ 3 (‘severe’ or worse) experienced statistically significant and clinically meaningful deterioration in overall QOL based on LASA scale. Additionally, BMI and hypofractionation impact overall QOL.

Our study supports the assertion that single item measures of QOL, like LASA, are useful in breast cancer and correlate well with PRO-CTCAE. Patients who reported grade ≥ 3 (‘severe’ or worse) PRO-CTCAE were found to have a 10-point drop in their overall QOL LASA score (p < 0.0001). As noted, a potential disadvantage of single-item measures, like LASA, is the lack of detail of the factors contributing to a decrease in quality of life [6]. The additional information provided from PRO-CTCAE can help clinicians identify the contributing factors of the decrease QOL that would not be available if LASA was used by itself. This also indicates that LASA may be useful as a screening tool. For example, all patients could be asked overall QOL LASA before each visit and if it indicates a worsening QOL then additional PRO measures like PRO-CTCAE could be used to better define the cause of the worsening QOL. This would help minimize the burden of long PRO questionnaires to patients while at the same time remaining useful for clinicians to improve patient care. Another concern is the potential for non-normal distribution of QOL using LASA including floor and ceiling effects. However, research has shown that the utilization of normal distribution theory is acceptable for Likert scale PROs even with floor/ceiling effects [15].

Hypofractionation has become a standard option for breast radiation therapy based on the results of several large randomized trials [16–18]. These trials have demonstrated less toxicity with hypofractionation compared to conventional fractionation [16, 17]. Additionally, hypofractionation is more cost effective compared to conventional fractionation [19]. As a result, the utilization of hypofractionation has become a standard and its use is increasing [20]. In keeping with this, the majority of patients in this study were treated with hypofractionation and were more likely to have better quality of life compared to women treated with conventional fractionation. This is similar to an analysis by Arsenault et al. who reported acute toxicity and PROs in a large randomized trial of conventional radiation versus hypofractionation following breast-conserving surgery for breast cancer [21]. A Breast Cancer Questionnaire, which is a validated instrument measuring quality of life in breast cancer patients, at baseline and 4 weeks after radiation was utilized. Patients randomized to hypofractionation had improved overall quality of life and quality of life attributed to less skin side effects, breast side effects, and improved attractiveness (all p < 0.01). Additionally, hypofractionated patients experienced less acute toxicity. Improved quality of life with hypofractionation in this study and ours furthers supports its use in the setting of breast cancer.

In the current study, higher BMI predicted for worse quality of life. Higher BMI has been shown to be associated with worse quality of life in other cancers [22, 23]. Similarly, higher BMI has also been shown to correlate to worse physician reported adverse events [24, 25]. In a study of breast cancer survivors, Anbari et al. used the validated 36-Item Short Form Health Survey to compare quality of life to changes in BMI which showed that a BMI change corresponded to 5 of the 8 domains [26]. However, increasing BMI corresponded to worsening quality of life in only 3 domains including physical functioning (p = 0.0052), role limitations related to emotional problems (p = 0.0216), and energy/fatigue (p = 0.0045). Interestingly, 2 domains, role limitations related to physical functioning and social functioning improved as BMI increased (p = 0.0052 and p = 0.0014, respectively). These studies suggest the relationship between a patient’s BMI and quality of life is complex and impacts quality of life during treatment and in survivorship. Additional research is needed to better define and explain the interaction.

Other studies have showed that single-item measures correlate well with multi-item measures [10, 27]. For example, Locke et al. investigated single-item LASA versus multi-item in patients with high grade gliomas [10]. Patients were assessed with LASA, the Functional Assessment of Cancer Therapy-Brain (FACT-Br), Profile of Mood States-Short form (POMS-SF), and Symptoms Distress Scale (SDS). They showed that LASA strongly correlated with each of the multi-item instruments and concluded that it might be useful as a screening measure with a general view of quality of life is needed. Similarly, Hubbard et al. performed a survey of providers after implementation of single-item LASA measures for fatigue, pain, and overall quality of life in clinical oncology practices [28]. The majority of providers in the study reported that the single-item measures enhanced their practice and did not change the length of clinic visits. The authors concluded that the study showed that single-item PROs measures can be used in oncology clinics with positive implications for patients and physicians.

This study has several limitations. First, although the LASA and PRO-CTCAE questionnaires were collected prospectively in an institutional registry, there was decreasing questionnaire responses over time. Part of this is likely due to patients being lost to follow-up and due to the extra burden of filling our questionnaires. Furthermore, additional follow-up is needed to evaluate how PROs evolve over time. The study is also subject to selection bias as to which patient filled out the questionnaires in follow up. It is possible that patients were more likely to fill out the questionnaires if they experienced worse outcomes. Lastly, this study was performed at a single academic institution, thus limiting its applicability to other practices.

In conclusion, in patients treated with curative intent radiation for breast cancer, the single-item LASA scale correlated well with PRO-CTCAE and supports the assertion that single-item overall QOL LASA could be used as a screening tool for PROs. Patients who reported a worsening PRO-CTCAE item to ‘severe’ or worse experienced statistically significant and clinically meaningful deterioration in overall QOL based on LASA. Hypofractionation appears to improve overall QOL while higher BMI predicted for worse QOL. These results contribute to the understanding of PRO-CTCAE for breast cancer patients and promote the value of integration of PRO-CTCAE items into future clinical trials.

## Data Availability

We have full control over the primary data and will allow it to be reviewed if requested.

## Abbreviations

AE: Adverse event
BC: Breast cancer
BIC: Bayes’ information criteria
BMI: Body mass index
FACT-Br: Functional Assessment of Cancer Therapy-Brain
LASA: Linear Analogue Self-Assessment
POMS-SF: Profile of Mood States-Short form
PRO-CTCAE: Patient-Reported Outcomes version of the Common Terminology Criteria for Adverse Events
PRO: Patient-reported outcome
QOL: Quality-of-life
RT: Radiation therapy
SDS: Symptoms Distress Scale
UVA: Univariate
